# Pediatric robotic surgery: issues in management—expert consensus from the Italian Society of Pediatric and Neonatal Anesthesia and Intensive Care (SARNePI) and the Italian Society of Pediatric Surgery (SICP)

**DOI:** 10.1007/s00464-022-09577-0

**Published:** 2022-09-19

**Authors:** Simonetta Tesoro, Piergiorgio Gamba, Mirko Bertozzi, Rachele Borgogni, Fabio Caramelli, Giovanni Cobellis, Giuseppe Cortese, Ciro Esposito, Tommaso Gargano, Rossella Garra, Giulia Mantovani, Laura Marchesini, Simonetta Mencherini, Mario Messina, Gerald Rogan Neba, Gloria Pelizzo, Simone Pizzi, Giovanna Riccipetitoni, Alessandro Simonini, Costanza Tognon, Mario Lima

**Affiliations:** 1 Division of Anesthesia, Analgesia, and Intensive Care, Santa Maria della Misericordia University Hospital, Perugia, Italy; 2 Pediatric Surgery, Department of Women’s and Children’s Health, University of Padua, 35128 Padua, Italy; 3 Department of Pediatric Surgery, IRCCS San Matteo Polyclinic, University of Pavia, Pavia, Italy; 4 Pediatric Surgery Unit, Federico II University of Naples, Naples, Italy; 5 Anesthesia and Intensive Care Unit, IRCCS Sant’Orsola Polyclinic, Bologna, Italy; 6 Pediatric Surgery Unit, Salesi Children’s Hospital, Polytechnical University of Marche, Ancona, Italy; 7 Department of Neurosciences, Reproductive and Odontostomatological Sciences, Federico II University of Naples, Naples, Italy; 8 Pediatric Surgery Unit, IRCCS Policlinico Sant’Orsola, University of Bologna, Bologna, Italy; 9 Institute of Anesthesia and Intensive Care, IRCCS A. Gemelli University Polyclinic Foundation, Sacred Heart Catholic University, Rome, Italy; 10 Pediatric Anesthesia, Department of Women’s and Children’s Health, Padua University Hospital, Padua, Italy; 11 Anesthesiology and Intensive Care Unit, Fondazione IRCCS San Matteo Polyclinic, Pavia, Italy; 12 Division of Pediatric Surgery, Santa Maria Alle Scotte Polyclinic, University of Siena, Siena, Italy; 13 Department of Pediatric Anesthesia and Intensive Care, Salesi Children’s Hospital, Ancona, Italy; 14 Pediatric Surgery Department, Vittore Buzzi’ Children’s Hospital, Milan, Italy; 15 Department of Biomedical and Clinical Science, University of Milan, Milan, Italy

**Keywords:** Pediatric robotically assisted surgery, Anesthesiology, Pediatric minimally invasive surgery, Consensus

## Abstract

**Background:**

Pediatric robotic-assisted surgeries have increased in recent years; however, guidance documents are still lacking. This study aimed to develop evidence-based recommendations, or best practice statements when evidence is lacking or inadequate, to assist surgical teams internationally.

**Methods:**

A joint consensus taskforce of anesthesiologists and surgeons from the Italian Society of Pediatric and Neonatal Anesthesia and Intensive Care (SARNePI) and the Italian Society of Pediatric Surgery (SICP) have identified critical areas and reviewed the available evidence. The taskforce comprised 21 experts representing the fields of anesthesia (*n* = 11) and surgery (*n* = 10) from clinical centers performing pediatric robotic surgery in the Italian cities of Ancona, Bologna, Milan, Naples, Padua, Pavia, Perugia, Rome, Siena, and Verona. Between December 2020 and September 2021, three meetings, two Delphi rounds, and a final consensus conference took place.

**Results:**

During the first planning meeting, the panel agreed on the specific objectives, the definitions to apply, and precise methodology. The project was structured into three subtopics: (i) preoperative patient assessment and preparation; (ii) intraoperative management (surgical and anesthesiologic); and (iii) postoperative procedures. Within these phases, the panel agreed to address a total of 18 relevant areas, which spanned preoperative patient assessment and patient selection, anesthesiology, critical care medicine, respiratory care, prevention of postoperative nausea and vomiting, and pain management.

**Conclusion:**

Collaboration among surgeons and anesthesiologists will be increasingly important for achieving safe and effective RAS procedures. These recommendations will provide a review for those who already have relevant experience and should be particularly useful for those starting a new program.

The advantages of minimally invasive, or laparoscopic, surgery (MIS) over open surgery include less trauma and blood loss, fewer postoperative complications, less pain, shorter hospital stays, and improved cosmetic outcomes [1, 2]. Incorporating robotic assistance can, furthermore, improve accuracy and precision, by eliminating operator tremor, thereby extending the indications of MIS to include complex procedures that would otherwise require open surgery. Robotically assisted surgery (RAS) is safe and appropriate for pediatric procedures that frequently require fine dissection and suturing in confined anatomical spaces [3, 4]. Accordingly, RAS has increasingly been adopted in several pediatric fields [5, 6]. Pyeloplasty and fundoplication are the RAS procedures most frequently performed in pediatric patients, whereas the most common complex reconstructive procedures include ureteral reimplantation and removal of choledochus cysts [6–10].

However, the expansion of RAS in pediatrics has faced some limitations. One challenge has been the reduced anatomical working space, which can limit the mobility of robotic instruments [8, 11]. The evolution of the instruments has partially overcome these limits [12], but careful patient selection remains an issue for the safe and successful use of robotic technology in the pediatric population.

The application of RAS in pediatric patients has increased rapidly in recent decades [13]; however, consensus guidelines are still lacking. For this reason, the Italian Society of Pediatric and Neonatal Anesthesia and Intensive Care (SARNePI) and the Italian Society of Pediatric Surgery (SICP) have organized a joint consensus taskforce to prepare such documentation.

## Materials and methods

This consensus is a collaborative initiative of Italian Society of Pediatric and Neonatal Anesthesia and Intensive Care (SARNePI) and Italian Society of Pediatric Surgery (SICP), who appointed a 21-membre Expert Task Force from ten clinical centers performing pediatric RAS in the Italian cities of Ancona, Bologna, Milan, Naples, Padua, Pavia, Perugia, Rome, Siena, and Verona. In December 2020 a first meeting was held to define the scope of the project, identify key issues, and agree consensus methods. It was decided that the focus should be on patients less than 18 years old, weighting more than 10 kg, with an American Society of Anesthesiologists (ASA) Classification of I–III, who were undergoing elective surgery of the thoracic, abdominal, or retroperitoneal region. Three main areas for investigation were identified (preoperative, intraoperative, and postoperative care), and corresponding subcommittees appointed. Within these phases, the panel agreed to address the areas listed in Table 1.

**Table 1:** The 18 critical areas addressed by the consensus group for the perioperative period

Phase	Issue
Preoperative	Patient selection
	Risk stratification
	ERAS
Intraoperative	Patient positioning
	Patient access
	Surgical times
	Pneumoperitoneum and ventilation
	strategies
	Hemodynamic changes and fluid therapy
	Hypothermia
	Anesthetic technique, depth monitoring, neuromuscular block
	Working space
	Role of the nursing staff
	Antibiotic prophylaxis
	Safety
Postoperative	Drains
	Postoperative analgesia
	PONV
	Thromboprophylaxis

*ERAS* Enhanced recovery after surgery, *PONV* Postoperative nausea and vomiting

Based on a literature review, the experts summarized the evidence and assembled a list of candidate statements with supporting evidence for each topic. Key issues were discussed during the second meeting in March 2021 and the document was finalized in a third meeting in April, after which the document was circulated, and subjected to two rounds of revision.

A modified Delphi approach was used to achieve consensus. The panel adopted three types of statement for the consensus document: statements of fact, evidence-based recommendations, and ‘best practice’ recommendations, the latter being defined as recommendations that the panel judged useful or needed, but for which there is only indirect supporting evidence. The panel graded the quality of evidence (Table 2) and strength of recommendation (Table 3) using the US. Preventive Services Task Force (USPSTF) system [14]. Statements for which consensus was achieved (> 70% agreement) were than resubmitted to the Experts at a Consensus Conference in September 2021: recommendations and supporting evidence were reviewed and discussed by the entire group, to achieve a final consensus (defined as > 70% agreement with < 15% disagreement). After the consensus meeting a draft report was prepared and circulated via email among all task force members. All Authors approved the final version as a condition for its acceptance.

**Table 2:** US preventive services task force grading of strength of recommendations [14]

Grade	Definition	Suggestions for practice
A	The USPSTF recommends the service. There is high certainty that the net benefit is substantial	Offer or provide this service
B	The USPSTF recommends the service. There is high certainty that the net benefit is moderate or there is moderate certainty that the net benefit is moderate to substantial	Offer or provide this service
C	The USPSTF recommends selectively offering or providing this service to individual patients based on professional judgment and patient preferences. There is at least moderate certainty that the net benefit is small	Offer or provide this service for selected patients depending on individual circumstances
D	The USPSTF recommends against the service. There is moderate or high certainty that the service has no net benefit or that the harms outweigh the benefits	Discourage the use of this service
I	The USPSTF concludes that the current evidence is insufficient to assess the balance of benefits and harms of the service. Evidence is lacking, of poor quality, or conflicting, and the balance of benefits and harms cannot be determined	Read the clinical considerations section of USPSTF Recommendation Statement. If the service is offered, patients should understand the uncertainty about the balance of benefits and harms

© US. Preventive services task force. September 2017

**Table 3:** Grading of quality of evidence (modified from US preventive services task force) [14]

Level of certainty*	Description
High	The available evidence usually includes consistent results from well-designed, well-conducted studies in representative primary care populations. These studies assess the effects of the preventive service on health outcomes. This conclusion is therefore unlikely to be strongly affected by the results of future studies
Moderate	The available evidence is sufficient to determine the effects of the preventive service on health outcomes, but confidence in the estimate is constrained by such factors as:
	• The number, size, or quality of individual studies
	• Inconsistency of findings across individual studies
	• Limited generalizability of findings to routine primary care practice
	• Lack of coherence in the chain of evidence
	As more information becomes available, the magnitude or direction of the observed effect could change, and this change may be large enough to alter the conclusion
Low	The available evidence is insufficient to assess effects on health outcomes. Evidence is insufficient because of:
	• The limited number or size of studies
	• Important flaws in study design or methods
	• Inconsistency of findings across individual studies
	• Gaps in the chain of evidence
	• Findings not generalizable to routine primary care practice
	• Lack of information on important health outcomes
	More information may allow estimation of effects on health outcomes

*The USPSTF defines certainty as ‘likelihood that the USPSTF assessment of the net benefit of a preventive service is correct.’ The net benefit is defined as ‘benefit minus harm of the preventive service as implemented in a general, primary care population’. The USPSTF assigns a certainty level based on the nature of the overall evidence available to assess the net benefit of a preventive service

© US. Preventive services task force. September 2017

## Results

### Preoperative phase

#### Patient selection

Given the constraints imposed by the robotic instruments and potential anatomical space limitations of the patient, the use of RAS in patients less than 1 year old or weighing less than 10 kg remains especially challenging, although there are reports of RAS being performed on patients weighing less than 7 kg [6, 8, 12, 15, 16]. There is currently no consensus on pediatric patient selection for RAS and there are no established parameters to guide this decision [8].

Due to the high cost of RAS, it has been applied mainly in complex pediatric reconstructive procedures such as pyeloplasty, fundoplication, ureteral reimplantation and choledochus cyst removal, and less frequently in simpler procedures such as varicocelectomy or appendicectomy [6–10, 16]. Robotic operating rooms (ORs) are often shared by several specialties, including adult surgery, and therefore may be located outside of pediatric hospitals or departments [6].

##### Statements

1. Robotic surgery in pediatric patients is recommended for complex procedures [6–10, 12, 15, 17–19] (Grade A—Level High)
2. Robotic surgery can be considered mainly in patients weighing more than 10 kg and older than one year [8, 9, 12, 15, 16, 18, 20–23] (Grade A—Level High)
3. Based on the experience at individual centers, robotic surgery can also be performed in selected patients of lower weight or age [8, 9, 12, 15, 18, 20–23] (Grade C—Level High)
4. Despite the need for a more complex organization, there are no contraindications to performing robotic surgery in facilities outside of pediatric centers [6] (Grade C—Level High)

#### Risk stratification

Pre-anesthetic evaluation identifies co-morbidities that may affect physiologic response to changes resulting from the pneumoperitoneum and tolerance to surgery [24]. Potential congenital anomalies, especially in the respiratory, nervous, and cardiovascular systems, should be considered and investigated, because these may be aggravated by pneumoperitoneum [1]. Intra-abdominal pressure (IAP) and absorption of CO_2_ during MIS are the major determinants of cardio-respiratory changes. Concerns that these influences could cause hypoxemia and pulmonary hypoperfusion had discouraged the use of MIS in children with heart disease; however, studies investigating the tolerability of IAPs in children with congenital heart disease (CHD) have established that IAPs between 8 and 12 mmHg in children less than 5 years old are safe, regardless of pre-existing conditions [25]. While the evidence does not indicate an absolute contraindication to MIS for patients with CHD [25–29], those with severe disease should undergo monitoring with transesophageal echocardiography, and pediatric cardiac anesthesia personnel should be involved with their pre-surgical evaluation and perioperative management [30].

A steep and prolonged Trendelenburg position causes an increase in central venous pressure (CVP) and therefore intraocular pressure (IOP); this compromises the outflow of aqueous humor into the episcleral venous circulation with consequent reduction of vision and the onset of optic neuropathy [31, 32]. Likewise, in elderly patients, an increase in intracranial pressure, measured indirectly with ultrasonographic measurement of optic nerve sheath diameter, is associated with delayed emergence from anesthesia, delirium, and postoperative cognitive impairment [33]. However, studies aimed at analyzing the predisposing factors for the increase in CVP (e.g., high values of positive end-expiratory pressure (PEEP) and peak pressures, hypercapnia, and decurarization) did not show critical increases in ocular and intracranial pressures in patients with no pre-existing ocular disease or brain pathology [32, 34]. The presence of diseases associated with an increase in ocular pressure (e.g., glaucoma) and intracranial pressure (e.g., neoformations, cerebral hemorrhage) does not, therefore, exclude them as an independent risk factor for severe complications [35].

##### Statements

1. When assessing suitability for robotic surgery in patients with comorbidities, stratification of the anesthetic risk by medical history, clinical examination, and diagnostic investigations is recommended [24, 36–39] (Grade A—Best Practice)
2. The presence of congenital heart disease does not constitute an absolute contraindication to robotic surgery, as established by clinical studies in other laparoscopic approaches [25, 26, 28, 40] (Statement of Fact)
3. Perioperative management and assessment of surgical timing for the frailest patients must be carried out by a multidisciplinary team of pediatric specialists [30] (Grade A—Best Practice)
4. In the adult setting, steep Trendelenburg position has been associated with very rare, serious ocular complications [31, 35, 41]; however, there is no evidence of this occurring in pediatric patients (Statement of Fact)
5. In patients with childhood glaucoma, it is recommended that intraocular pressure be stabilized before robotic surgery [42, 43] (Grade A—Level Moderate)

#### Enhanced recovery after surgery

Enhanced Recovery After Surgery (ERAS) is a multimodal, multidisciplinary, evidence-based approach to promote faster post-operative recovery [44]. Enhanced Recovery After Surgery guidelines promote the use of MIS; in relation to this, RAS is widely used for pediatric gastrointestinal surgery [45–47], where it can reduce costs, length of stay, and complication rates. A single center study on the implementation of ERAS for pediatric colorectal surgery demonstrated a significant decrease in the median length of hospital stay with no increase in rates of complication or readmission [46].

##### Statements

1. The adoption of a suitable ERAS (Enhanced Recovery After Surgery) program reduces the direct costs of robotic surgery and promotes its economic sustainability [48] (Grade A—Level High)
2. Every center conducting robotic surgery should implement an enhanced recovery program based on the most recent evidence for each type of pediatric robotic surgery [16, 49–51] (Grade B—Level Moderate)

### Intraoperative phase

#### Patient positioning

Establishing the correct position of the patient is a dynamic process, managed by the surgeon and the anesthetist, which must optimize the visibility of the surgical field, give the anesthetist access to the patient, and minimize the development of complications (e.g., compression injuries). Adequate padding is required on and around the face and pressure points to avoid skin, soft tissue, and nerve injuries [1, 52, 53].

##### Statements

1. When applying patient restraint systems on the operating table, particular attention to the following is recommended:
  Ensure that the endotracheal tube is correctly applied, and the head is protected
  Use mattresses that prevent slipping
  Place arms preferably along the body
  Apply eye protection
  Apply anti-decubitus aids to prevent nerve injuries (e.g., heel and elbow pads, popliteal support positioners, pillows) [1, 52, 53] (Grade A—Level High)
2. It is advisable to keep one arm freely accessible to the anesthesiologist, whenever possible [1, 52, 53] (Grade B—Level High)

#### Patient Access

The number and type of peripheral vascular access points required during robotic surgery depends on the type of surgery [1, 3, 5], as well as the patient’s age, weight, and clinical condition [54–56]. The situation will also depend on the patient’s vascular history and the manual skills of the anesthesiologist. Prior to surgery, the access points (venous and peripheral) must be properly fixed and controlled, given the potential difficulty of accessing the patient after docking [3]. Inadequate attachment can cause damage to the cannulated vessel wall, malfunction, erosion, inflammation, thrombosis, occlusion, and exit-site infections. Sutureless adhesive or subcutaneous fixation and anchoring systems are effective and safe [57–59], and there is no strong evidence to suggest that one system works better than another [57].

Central venous access, while not always necessary, can be useful and advantageous in pediatric RAS. Positioning the line is not without risks, however, and the decision must be based on specific circumstance, such as the need for frequent blood sampling, or the administration of hyperosmolar fluids, antibiotic therapy, or vasoactive drugs [60–62]. Ultrasound-guided line placement can reduce the risk of complications and optimize positioning [60, 61]. While the internal jugular vein is the most frequent site for positioning a central venous line via ultrasound, this approach is difficult in infants and very young children [62]. Useful, and readily visible, alternatives to use with ultrasound include the supraclavicular approach to the subclavian vein, the brachiocephalic veins or the axillary vein, which tend to remain open regardless of hemodynamic status or stage of respiratory cycle [60, 61]. Placement of an arterial catheter is an optional, advanced step that allows both continuous blood pressure (BP) monitoring and serial blood gas analysis [63].

Placement of a nasogastric tube before surgery enables decompression of the stomach, which is frequently inflated during the induction of anesthesia [3, 64, 65]. Decompression is critical in abdominal and urological RAS, because it improves the visibility of the operative field and minimizes the risk of accidental gastric damage [63, 65, 66].

Bladder catheter placement is essential for fluid management, monitoring urinary output during surgery [65, 66], and the avoidance of bladder damage during the placement of trocars in abdominal surgery [64, 65].

##### Statements

1. Vascular access must be established prior to docking [1, 3, 54, 63, 65] (Grade A—Level High)
2. It is good clinical practice to place at least two peripheral lines and, especially if there is a high risk of intraoperative bleeding, one central access line [1, 3, 54, 63, 65] (Grade B—Level High)
3. Ensuring that the infusion lines are of adequate length and free of kinking / obstructions, and that the taps are easily accessible, is recommended [1, 3, 54, 63, 65] (Grade A—Level High)
4. The optimal aids to fix vascular access points and minimize the risk of dislocation are sutureless, adhesive or subcutaneous systems [57, 58, 67–69] (Statement of Fact)
5. Positioning of an arterial line should be assessed on the basis of the patient's clinical condition and the details of the intervention [3, 63, 70] (Grade A—Level High)
6. Intraoperative gastric tube placement is required [3, 63–66] (Grade A—Level High)
7. Bladder catheterization, when indicated, must be placed before surgery [3, 63–66, 70] (Grade A—Level High)

#### Surgical times

While consideration of procedure time is important for any surgery, timing takes on added importance with pediatric RAS because many preparatory procedures are performed after induction, increasing the length of anesthesia [10, 53, 71, 72]. Precise definition of procedure time, and training, are the key factors toward timing optimization [73, 74]. Standardizing and repeating the interventions improves patient management [10, 74, 75]. Docking time (i.e., approaching the robot, positioning and anchoring the ports) is a crucial area for training, and in pediatric patients it is better to define procedure time as starting from first incision (the knife-to-skin) to completion of docking [53, 71, 72]. Positioning must take into consideration the patient’s age, size, and pathology. Any potential maneuvers (e.g., endoscopic) must be considered during intervention planning and the preoperative brief [72, 75, 76].

##### Statements

1. In the pediatric patient, it is better to consider knife-to-skin time rather than docking. 20 min is considered a good time [10, 71, 72] (Statement of Fact)
2. Docking must be jointly performed by doctors and nurses during the training period (up to complete autonomy) together with specialized technicians [53, 72, 73] (Grade A—Level High)
3. Marking the position of the surgical access ports reduces time and facilitates the procedures [53, 75] (Statement of Fact)
4. The use of additional instruments during robotic surgery (e.g., gastroscope, colonoscope, cystoscope) requires preemptive patient preparation and positioning [76, 77] (Statement of Fact)

#### Pneumoperitoneum and ventilation strategies

A prospective single-blind randomized study conducted in infants less than 10 kg undergoing pneumoperitoneum for laparoscopic renal surgery showed that an insufflation pressure between 6 and 8 mmHg provides excellent surgical conditions with minimal physiologic impact [78]. Transperitoneal insufflation pressures up to 10 mmHg do not induce significant hemodynamic changes [9, 71, 79], while insufflation pressures greater than 10 mmHg do not increase workspace in infants [64]. Pressures up to 12 mmHg have been reportedly well tolerated in patients aged 8–16 years [80].

In the event of intraoperative hypoxia, alveolar recruitment maneuvers should be performed only after excluding other causes, such as displacement of the endotracheal tube [63]. Recruitment is associated with a high risk of lung trauma and should be performed only after adjusting FIO_2_ in correlation with SaO_2_ or with PaO_2_, if available. The risk is lower when protective ventilation is used [81]. Greater absorption of CO_2_ in very young patients requires a high respiratory rate to eliminate CO_2_ and reduce the risk of hypercapnia; this risk may be higher if volume-controlled ventilation is used (target volume 6–7 ml/kg) with the I:E ratio increased or reversed.

Using a positive end-expiratory pressure (PEEP) greater than 5 cm H_2_O should provide for the recruitment of atelectatic lung areas [82]. It may be useful to calculate the PEEP based on the pressure/volume curve.

Pulmonary ultrasound allows intraoperative assessment of atelectatic lung areas. This advanced monitoring technique was used in a randomized controlled trial performed in a pediatric population undergoing laparoscopy; results showed that alveolar recruitment maneuvers followed by PEEP application performed immediately after anesthetic induction, and before onset of carboperitoneum, can prevent atelectasis [83, 84].

To prevent atelectotrauma in pediatric patients, studies suggest using protective ventilation with a tidal volume of 6–7 ml/kg, peak pressures below 28 cm H_2_0, and a PEEP of 5 cm H_2_O [81, 85]. No studies have investigated correlations between ventilation mode and perioperative outcomes in pediatric surgery. Studies conducted in adults undergoing pneumoperitoneum with Trendelenburg positioning have shown that a PEEP of 10 cm H_2_0, or 15 cm H_2_0 applied after alveolar recruitment maneuvers, results in a greater distribution of intraoperative alveolar ventilation, compared with standard PEEP at 5 cm H_2_O although with no impact on postoperative outcome [86, 87]. In the pediatric population with acute respiratory distress syndrome, use of inverse ratio ventilation did not substantially improve oxygenation and reduced CO_2_ elimination [88]. Volume targeted pressure-controlled ventilation mode is optimal for pediatric patients undergoing RAS, including patients of low weight, because it can deliver very low tidal volumes [81].

##### Statements

1. It is recommended that pneumoperitoneum pressure be maintained in the following ranges [9, 53, 63, 64, 78, 79, 89–92]:
  < 2 years old: 6–10 mmHg
  2–10 years old: 10–12 mmHg
  10 years old: 12 mmHg (Grade A—Level High)
2. The use of protective ventilation (tidal volume 6–7 ml/kg and lowest possible driving pressure) is recommended to obtain optimal SaO2 with the minimum FIO2 and acceptable pCO2 values [63, 81] (Grade A—Moderate)
3. In case of insufficient gas exchange and/or suspicion of atelectatic areas, proceeding with alveolar recruitment via the use of PEEP (between 5 and 10 cm H2O) is recommended [82, 83, 93] (Grade A—Moderate)
4. It is recommended to perform alveolar recruitment maneuvers after adjusting FIO2 relatively to SaO2 or, if available, relatively to PaO2 [82, 83, 93] (Grade A—Moderate)

#### Hemodynamic changes and fluid therapy

Background infusion can be 10 ml/kg/hr of an isotonic polyelectrolyte solution containing 1–2.5% glucose, possibly buffered [94, 95]. Pediatric patients treated with restrictive fluid replacement (5 ml/kg) during major abdominal surgery require additional boluses to ensure hemodynamic stability and acid–base control [96], highlighting the need to maintain extracellular volume in these patients. This is especially true for infants, where the extracellular volume is larger [97].

During more complex surgery, and in patients with hemodynamic instability, volume replacement with boluses of 10–20 ml/kg of buffered polyelectrolyte solutions without glucose should be considered until hemodynamic stability is achieved, repeated up to three times to avoid fluid overload [98]. Consider administering blood products. The infusion regimen can be adjusted according to the duration of surgery, blood loss, blood glucose levels, and acid–base balance [98].

Guidelines on preoperative fasting recommend its minimization and encouraging pediatric patients to drink clear fluids up to one hour before surgery; postoperative fasting should also be reduced to the minimum required [99]. When it is not possible to maintain euvolemia in the preoperative period, volume replacement should be administered before anesthetic induction. Volume maintenance with fasting follows the 4-2-1 rule multiplied by the hours of fasting [100].

Quantification of intraoperative fluid loss is quite empirical and must include perspiration and blood. Concerning insensible loss, perspiration can be considered collateral to the peritoneal absorption of CO_2_ which is inversely proportional to the age of the patient and, unlike in adults, fails to reach a plateau instead being incremental with the duration of surgery [101, 102].

During RAS, the risk of cerebral edema rises in relation to increased time-dependent absorption of CO_2_ and the use of the Trendelenburg position. The risk can be reduced by administering isosmolar polyelectrolyte solutions with plasma. The Trendelenburg position also increases the risk of airway edema, which can be minimized by maintaining euvolemia and avoiding fluid overload.

Hemodynamic changes from pneumoperitoneum are generally well tolerated in healthy pediatric patients, when physiological homeostasis is maintained [63]. Clinical monitoring of capillary refill, acid–base balance, especially base excess, the presence of lactate, and urinary output (> 1 ml/kg/hr), represent the basic level of monitoring. Advanced monitoring techniques may be added to the above although the use of hemodynamic ultrasound is technically impractical. Standard monitoring of vital parameters includes BP, continuous ECG, SaO_2_, and body temperature. Pediatric BP monitoring is not as indicative of change in cardiovascular status (i.e., cardiac output, stroke volume) as in the adult and should, therefore, not be relied upon alone for monitoring cardiac output. Invasive monitoring of arterial pressure and CVP may also provide information on ScVO_2_. Continuous monitoring of pediatric patients with arterial cannulation is essential due to the risk of massive bleeding following accidental disconnection. Since hemodynamic changes are more evident during hypovolemia, using a tool to assess fluid-responsiveness in patients mechanically ventilated at positive pressure may be appropriate [103].

Trendelenburg and anti-Trendelenburg positions can aggravate hemodynamic change. In particular, the Trendelenburg position can favor venous return which both increases cardiac output and cause cephalic displacement of the diaphragm, which can compromise ventilation and induce pulmonary atelectasis.

Changes in BP during the respiratory cycle in mechanically ventilated patients can indicate hemodynamic responsiveness to fluid load. Arterial waveform analysis can be used to monitor this if an intra-arterial cannula is in situ. In the pediatric population, plethysmography and ultrasound represent valid tools for non-invasive intraoperative hemodynamic assessment [63, 104–106].

##### Statements

1. Patients undergoing robotic surgery in euvolemia have a lower risk of hemodynamic changes induced by pneumoperitoneum with or without the Trendelenburg position [107, 108] (Statement of Fact)
2. A 10 ml/kg/hr background infusion of an isotonic polyelectrolyte solution, possibly buffered, containing glucose at a concentration of 1–2.5%, is recommended [63, 98] (Grade B—Level High)
3. To prevent hyponatremic conditions, the administration of glucose solutions that do not contain electrolytes is not recommended [98, 100, 106, 109] (Grade D—Level High)
4. In order to avoid hyperchloraemic acidosis from infusion of saline-based fluid 0.9%, the administration of buffered polyelectrolyte solutions is recommended [98, 100, 106, 109] (Grade B—Level High)
5. To avoid fluid overload, especially in infants and patients with cardiological/renal comorbidities, the use of infusion or syringe pumps is recommended [98] (Grade A—Best Practice)
6. In more complex interventions and/or fragile patients, invasive monitoring of peripheral arterial pressure (e.g., arterial vessel cannulation) and central venous pressure (e.g., a central venous catheter) are indicated, which can also provide information on ScVO_2_. Advanced hemodynamic monitoring is also recommended in these patients [60–63] (Grade A—Level High)

#### Hypothermia

Robotically assisted surgery exposes the patient to the risk of hypothermia; therefore, careful monitoring of central body temperature and application of appropriate systems for intra-operative warming are warranted [1, 5, 110, 111].

##### Statements

1. Body temperature should be closely monitored and intraoperative hypothermia avoided [1, 5, 110] (Grade A—Best Practice)
2. Use adequate body heating systems (e.g., forced air or water mattresses, administration of heated IV fluids) and maintain an adequate temperature in the operating room [1, 5, 110] (Grade A—Best Practice)
3. It is recommended to insufflate with heated gas and to keep the insufflation flow below 2 L/min [1] (Grade A—Level Low)
4. To counteract redistributive hypothermia, pre-warming of the patient for at least 10 min prior to induction is recommended [5] (Grade A—Best Practice)

#### Anesthetic technique, monitoring of anesthesia depth, neuromuscular block

Neuromuscular blockade (NMB) is used during RAS to ensure immobility and stabilize insufflation pressure. Rocuronium combined with cisatracurium blocks acetylcholine receptors and provides effective blockade [112, 113]. Monitoring of NMB using peripheral nerve stimulation (e.g., train of four) is essential to ensure correct dosing during induction and maintenance, and to monitor postoperative reversal [114, 115]. Complete reversal of NMB at the end of surgery is important in order to reduce the risk of post-operative residual curarization (PORC), because the latter is associated with an increased risk of postoperative pulmonary complications (PPC) [116, 117]. Neuromuscular blockade reversal can be achieved by administering a cholinesterase inhibitor such as neostigmine, which increases acetylcholine levels, or by administering sugammadex to sequester rocuronium. The occurrence of PORC may depend on the type of block and reversal agents used. The risk of postoperative respiratory complications is reduced with sugammadex [113, 118, 119]. Compared with neostigmine, sugammadex reverses rocuronium-induced NMB more quickly, regardless of anesthesia depth [118, 120], and is associated with a lower risk of respiratory and cardiovascular adverse events [121].

Monitoring anesthesia depth can help to avoid overuse of intraoperative anesthetic agents and facilitate faster, and more manageable, emergence [122]. The depth of anesthesia should be monitored with the bispectral index (BIS) [123]; however, evidence supporting its use in infants less than 6 months old is lacking [124].

Loco-regional anesthesia is often used in conjunction with general anesthesia (GA) for pediatric surgery [125, 126]. With regards to preference during RAS, there is no consensus between central or peripheral blocks, although some evidence leans toward peripheral transversus abdominis plane (TAP) block to better control pain and reduce the intra- and postoperative use of analgesics [127]. Caudal block for some urological surgeries, when indicated, may reduce the need for intraoperative anesthetic drugs and reduce post-operative nausea and vomiting (PONV) compared with TAP blockade or general anesthesia alone [125]. In pediatric patients undergoing MIS, the use of a locally infused anesthetic is as effective as intrathecal opioids for pain control, but avoids the potential complications associated with this route of administration [128].

Intra-operative pain management is important in RAS. The main causes of intra- and post-operative pain are the surgical incisions for trocar insertion and visceral pain caused by pneumoperitoneum [66]. A multimodal approach to pain control is recommended, when intravenous (IV) analgesics (i.e., opiates and NSAIDs) are associated with appropriate loco-regional anesthetic techniques [1, 66, 125, 129]. Combining these two techniques can control both abdominal wall and visceral pain [112, 113].

Local anesthetics have a membrane-stabilizing effect at the neuromuscular junction that acts in synergy with neuromuscular blockers to reduce lactic acidemia and attenuate bronchial hyper-reactivity [54]. The use of loco-regional anesthesia decreases the need for intraoperative opiate administration and its associated side effects, while improving patient outcomes [1, 125, 130].

##### Statements

1. NMB (neuromuscular blockade) is always indicated [118, 120, 121, 131] (Grade A—Best Practice)
2. Monitoring of NMB is essential for correct management of intra- (i.e., induction, maintenance) and postoperative (i.e., pharmacological reversal of NMB) curarization [118, 120, 121, 131] (Grade A—Best Practice)
3. NMB must always be antagonized at the end of surgery to avoid postoperative pulmonary complications associated with the presence of residual NMB [112, 113, 118] (Grade A—Best Practice)
4. Monitoring the depth of anesthesia is recommended [123, 132] (Grade A—Best Practice)
5. The use of loco-regional anesthesia is recommended to reduce the intra- and postoperative administration of opioid anesthetics and analgesics [66, 126, 127, 133–136] (Grade B—Level High)

#### Work space

High IAP is a major cause of hemodynamic instability during MIS, and low levels of hemodynamic change can be observed from a pressure of 12 mmHg [92]. In pediatric patients, insufflation and subsequent abdominal distension increase the risk of vagal reflexes or bradycardia [91]; therefore, gradual insufflation is recommended [92, 137]. In younger children, insufflation pressures ranging from 4 to 12 mmHg generally provide adequate operating space and a good view of the internal anatomical structures [138]. Working space in infants may be expanded slightly by retracting the ports by 1–2 cm after docking to ‘tent’ the abdominal wall [137, 139].

##### Statement

1. To reduce pressure and gain surgical space, the application of gentle traction on each trocar is recommended [6, 89, 139–142] (Grade B—Level High)

#### Role of the nursing staff

Nurses working as part of the RAS team must have a high of level professional skill and be offered a well-structured training program to ensure efficiency and maximum patient safety. Working as part of the surgical team, each nurse may be assigned a specific role, such as chief nurse, scrub nurse, or circulating nurse [143]. The development and use of standardized procedures and surgical checklists for each robotic procedure may improve patient safety and outcomes [143].

##### Statements

1. It is recommended that procedures and tools be standardized, also by preparing a specific checklist [53] (Grade A—Best Practice)
2. It is advisable to create a dedicated nursing team that includes three nurses in the operating room [6, 143–145] (Grade B—Level Low)
3. It is advisable to identify a single contact person among the nursing staff for taking charge of the patient in the room (compilation of the in and out check list) [6, 143–145] (Grade B—Level Low)
4. It is advisable to periodically schedule training courses [6, 143–145] (Grade B—Level Low)

#### Antibiotic prophylaxis

Antibiotic prophylaxis is indicated for clean–contaminated procedures, clean surgery in the case of prosthetic implants, or when the onset of infection may have serious or fatal consequences. However, in most cases antibiotic prophylaxis is not indicated for clean surgery [146–149]. The choice of prophylaxis may be guided by risk factors such as the ASA score, wound classification, and the duration of the intervention. Pediatric RAS of the pelvic, abdominal or thoracic regions considered clean–contaminated or contaminated, are indications for perioperative prophylaxis with an antibiotic agent that complies with local antimicrobial stewardship guidelines and microbiological surveillance, administered at adequate dosage, timing, and redosing, if indicated [146–148].

##### Statements

1. It is recommended to administer IV antibiotic prophylaxis in the operating room 30–60 min before the incision [146–149] (Grade A—Best Practice)
2. For prophylactic purposes, a single shot medium or high dose is recommended [146–149] (Grade B—Best Practice)
3. Administration of an additional intraoperative dose is recommended:
  If the surgery is still in progress after a twice the half-life of the drug
  If blood loss during surgery is ≥ 25 ml/kg [146–149] (Grade A—Best Practice)
4. It is recommended to continue with antibiotic therapy for the first 24 h post-surgery only in defined clinical situations when the risk index for postoperative infections is high [146–149] (Grade A—Best Practice)

#### Safety

Preoperative anxiety in pediatric patients is associated with significant negative clinical outcomes and emergence delirium; however, a variety of pharmacological (i.e., premedication) and non-pharmacological strategies to minimize anxiety exist [150–154]. Non-pharmacological strategies, including multimodal techniques or the presence of parents at induction, can be valid alternatives to drugs in many cases [150–154].

Operating room safety in RAS is compromised by the physical distance between the surgeon, console, and the rest of the team, with the patient. Communication within the team is essential for coordinating activities and preventing perioperative accidents. The World Health Organization has developed ‘Guidelines for Safe Surgery’ and the ‘Surgical Safety Checklist’ which are designed to improve patient safety and reduce postoperative complications [155]. A detailed surgical checklist should be adapted to the procedure and setting, and cover the procedures required prior to induction, before skin incision, and before releasing the patient to recovery [155].

Safety is also improved through timed training simulations to develop proficiency in docking/undocking and other critical procedures [156], as well as simulation using models to develop and maintain robotic surgical skills [157–159].

##### Statements

1. Pre-anesthesia is not imperative: pharmacological and non-pharmacological methods can be used to reduce patient anxiety [150–153] (Statement of Fact)
2. A briefing is recommended the day before surgery, while a surgical check list should be used prior to, during and immediately after the procedure (e.g., sign in, time out, sign out) [155, 160–164] (Grade A—Best Practice)
3. The debriefing should always be performed at the end of the procedure as part of the improvement process of the operating team to increase operating room safety [155, 160–164] (Grade C—Best Practice)
4. Before performing pediatric RAS, it is recommended that surgeons:
  Attend simulations and specialized courses
  Undergo practical training, e.g., ‘hands-on’ courses, exercises on inanimate or virtual and animal models
  Undertake docking and quick undocking simulations with technicians and RAS specialists, especially in the initial stages of training [156–159, 165, 166] (Grade A—Level High)

### Postoperative Phase

#### Drains

In pediatric RAS, drain tubes are generally not left in place; however, abdominal or thoracic drains could be retained if deemed necessary by the surgeon (e.g., because of unforeseen events) [167].

#### Statement

1. It is advisable to avoid the use of surgical drains when possible, and minimize their residency time when used [51, 167–169] (Grade B—Level High)

#### Postoperative analgesia

As with all surgery, postoperative pain must be managed carefully, beginning with the anesthesiologist upon anesthesia emergence and continuing with the nursing staff in the recovery area and ward. Postoperative pain should be monitored with validated, age-appropriate pain scales. In the absence of specific guidelines for RAS, the European Society for Pediatric Anaesthesiology (ESPA) pain committee guidance for postoperative pain management in children is considered valid [170]. Clinical and electronic monitoring standards will depend on age, comorbidities, extent, and complexity of the surgery, and use of sedative medications. Particular care is required and monitoring when administering opioid infusion to infants less than one year of age, and when continuous infusion is used [170].

Multimodal analgesia is recommended and may include a selection from the following drugs and/or procedures: paracetamol-ketorolac, morphine, tramadol-ibuprofen, possibly administered in combinations [129]. Corticosteroids may enhance postoperative pain relief, prolong the duration of regional anesthesia, and reduce postoperative nausea and vomiting [170].

#### Statements

1. Refer to the European Society for Pediatric Anaesthesiology (ESPA) pain committee guidance on the management of postoperative pain [170] (Grade A—Level High)
2. The use of a single intraoperative dose of dexamethasone is recommended [171–177] (Grade A—Level Moderate)

#### Postoperative nausea and vomiting

**Fig. 1:**
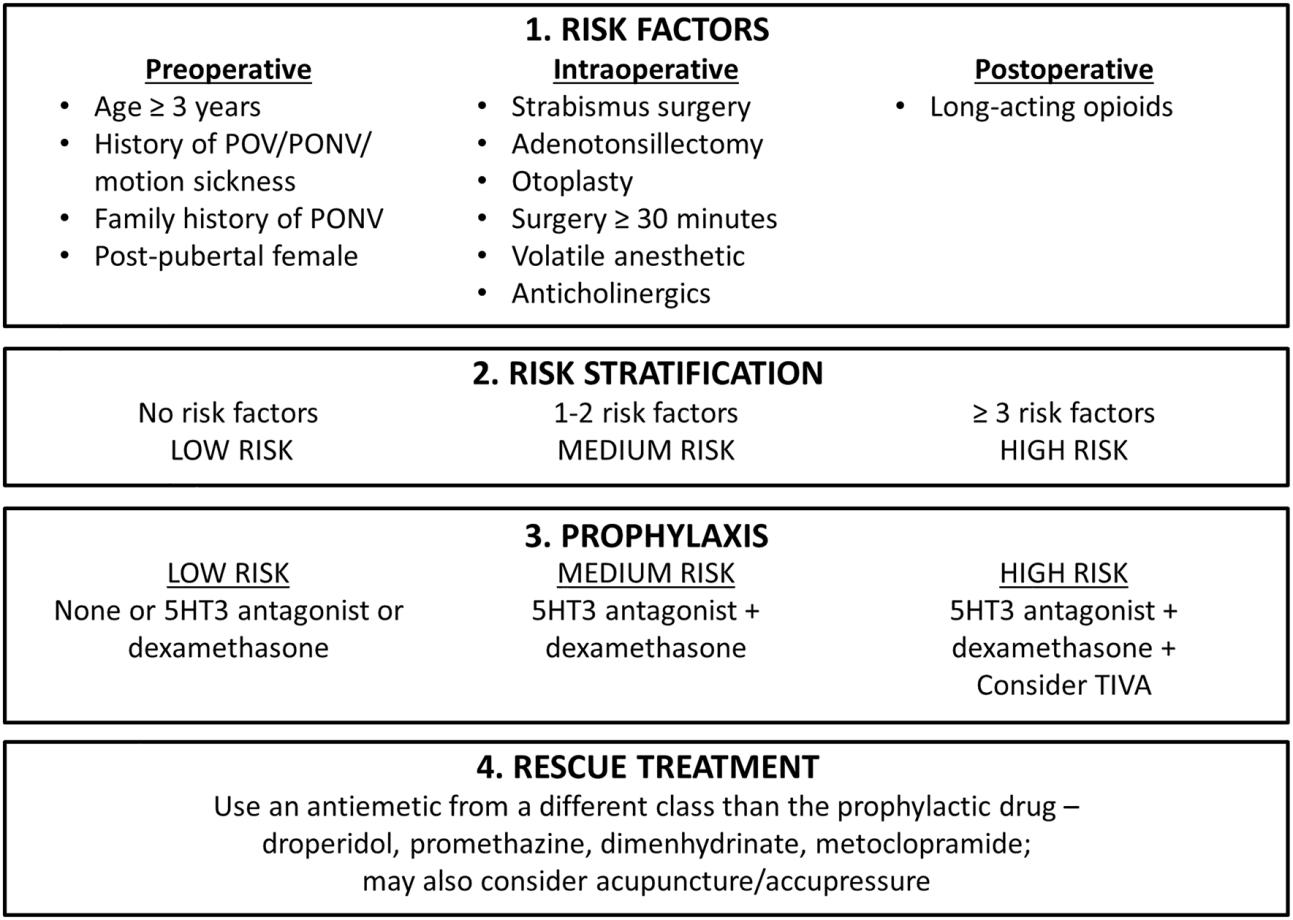
Summary of recommendations for POV/PONV management in children, including risk identification, risk-stratified prophylaxis, and treatment of established POV. *5-HT3* 5-hydroxytryptamine 3, *PONV* postoperative nausea and vomiting, *POV* postoperative vomiting, *TIVA* total IV anesthesia [179]. For permission requests, contact info@aserhq.org

The incidence of PONV in children undergoing laparoscopic cholecystectomy is approximately 39% [178]. Guidelines for managing PONV from the American Society of Enhanced Recovery and Society for Ambulatory Anesthesia provide evidence-based recommendations for pediatric patients (Fig. 1) [179]. A multimodal approach to PONV control should include preoperative risk evaluation and stratification, adequate IV fluid hydration, antiemetic prophylaxis, and pain management with opioid-sparing medications and regional anesthesia [180]. Postoperative opioid use is also a risk factor for nausea and vomiting [180]. Useful antiemetics for pediatric patients include dexamethasone or serotonin 5-hydroxytryptamine-3 receptor antagonists, with escalation to a combination of them (i.e., multimodal antiemetic therapy), and the use of propofol total IV anesthesia for children at high risk of PONV [179–181].

#### Statements

1. The use of the post-operative vomiting in children (POVOC) score is recommended [178–180] (Grade A—Best Practice)
2. The use of a prophylactic antiemetic protocol is recommended [169, 179, 182] (Grade A—Best Practice)
3. Rescue treatment with antiemetics of a class other than those used for prophylaxis is recommended [179, 180] (Grade A—Best Practice)

#### Thromboprophylaxis

**Fig. 2:**
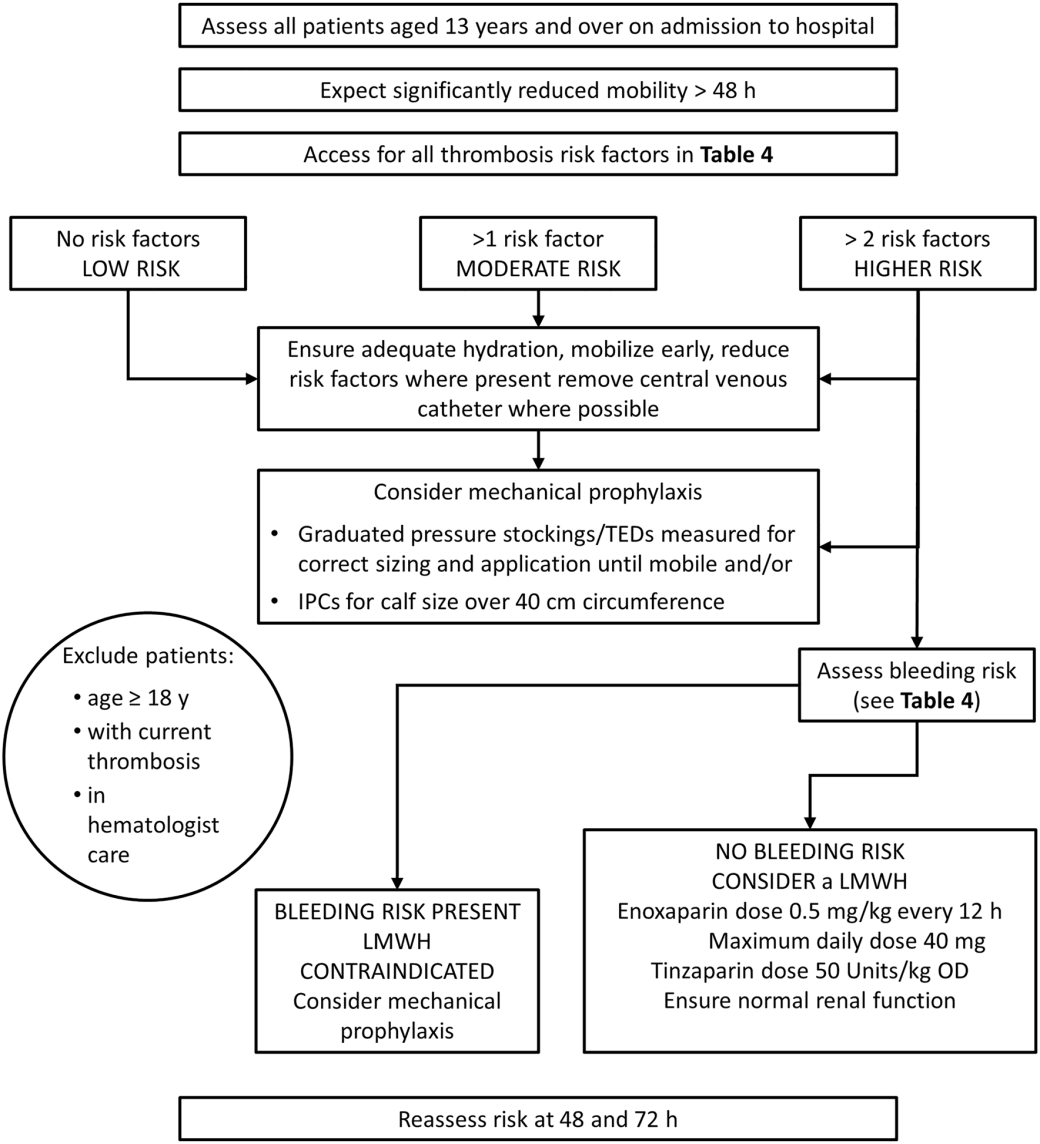
Risk assessment for venous thromboembolism for adolescents aged 13 years and older: decision-making algorithm from the Association of Paediatric Anaesthetists of Great Britain and Ireland (APAGBI). *IPC* intermittent pneumatic compression, *LMWH* low molecular weight heparin, *TEDs* thromboembolic deterrent stockings [187]. (Redrawn by permission from the APAGBI) [187]. [request permission: apagbiadministration@anaesthetists.org]

The low incidence of venous thromboembolism in pediatric surgical patients (approximately 0.2%) obviates the need for prophylaxis in patients without risk factors [183–185]. Accordingly, risk should be stratified [51, 185–187], and the Association of Paediatric Anaesthetists of Great Britain and Ireland (APAGBI) Guidelines include a risk assessment chart (Table 4) and decision algorithm (Fig. 2) to help with patient assessment [183, 187].

**Table 4:** Risk factors for venous thromboembolism in children, from the Association of Paediatric Anaesthetists of Great Britain and Ireland (APAGBI) Guidelines [187]

**Patient related**	**Admission related**
Bleeding risk^a, b^	
Acquired bleeding disorders (e.g., acute liver failure)	Neurosurgery, spinal surgery, or eye surgery
Untreated inherited bleeding disorders (e.g., hemophilia and von Willebrand’s disease)	Lumbar puncture/epidural/spinal anesthesia expected within the next 12 h
Concurrent use of anticoagulants known to increase the risk of bleeding (e.g., warfarin with INR > 2)	Lumbar puncture/epidural/spinal anesthesia within the previous 4 h
Thrombocytopenia	Active bleeding
Uncontrolled systolic hypertension (> 230/120 mmHg)	
Thrombosis risk^a^	
Central venous catheter	Significantly reduced mobility for 3 days or more
Active cancer or cancer treatment	Severe trauma with ISS > 9
Dehydration	Spinal cord injury with paralysis
Known thrombophilia	Total anesthetic + surgical time > 90 min
Obesity (BMI > 30 kg/m^2^)	Acute severe sepsis
≥ 1 significant medical comorbidity (e.g., congenital, or low output heart disease, sickle cell disease, metabolic or inflammatory conditions)	Surgery involving pelvis or lower limb with a total anesthetic + surgical time > 60 min
Personal history of VTE, first-degree relative with a history of VTE age < 40 years	Critical care admission intubated and ventilated
Estrogen-containing contraceptive therapy	Severe burns
Pregnancy or < 6 weeks post-partum	

^a^Clinicians may consider risks in addition to those listed

^b^If an increased risk of bleeding is documented in the risk assessment, thromboprophylaxis with low molecular weight heparin is relatively contraindicated

*BMI* Body mass index, *INR* international normalized ratio, *ISS* Injury Severity Score, *VTE* venous thromboembolism

Thromboprophylaxis should be started immediately after surgery, except in patients who need neuro-axial catheters for anesthesia, when it should be started no later than 12 h after catherization. If the patient’s risk profile dictates the use of thromboprophylaxis, it should be continued for 48–72 h, after which a new risk assessment should be undertaken [183].

#### Statement

1. Perioperative thromboprophylaxis is recommended in patients with confirmed thromboembolic risk factors or when prolonged immobilization is required [51, 183, 188] (Grade A—Level High)

## Conclusion

Consensus documents providing evidence-based recommendations for pediatric RAS are currently lacking. This multidisciplinary panel of experts has identified critical areas of concern regarding the preoperative, intraoperative, and postoperative phases of pediatric RAS, and formulated evidence-based guidelines. The proposed guidance covers all phases of pediatric RAS from the perspectives of anesthesiology and surgery. We addressed preoperative patient assessment and preparation, intraoperative patient management, in terms of operating room organization, patient preparation and positioning, the surgical procedure itself, and postoperative care, including pain management, drainage, realimentation, and hospital discharge, in order to establish a protocol that has to be followed by all RAS team members.

In future, given its advantages, the applications of pediatric RAS are likely to expand further and will follow the investment and technological development currently underway. This article will therefore be very useful for those who already have robotic surgical experience and, above all, anyone who plans to start a new program. In pediatric RAS, close collaboration between surgeons, anesthesiologists, and nurses will be increasingly important and necessary to achieve the objectives of safe surgical outcomes. Moving forward, the respective scientific societies will have the difficult task of supporting and conducting scientific efforts for this purpose.
